# Supplementary material for: Naturally Degradable Photonic Devices with Transient Function by Heterostructured Waxy‐Sublimating and Water‐Soluble Materials
Source: Adv Sci (Weinh). 2020 Sep 4;7(20):2001594. doi: 10.1002/advs.202001594 (PMC7578881; doi:10.1002/advs.202001594)
Supplement: Supplementary file 1 — Supporting Information [file ADVS-7-2001594-s001.pdf]

© 2020 Wiley-VCH GmbH

ADVANCED SCIENCE
Open Access

Supporting Information

for *Adv. Sci.,* DOI: 10.1002/advs.202001594

Naturally Degradable Photonic Devices with Transient Function by Heterostructured Waxy-Sublimating and Water-Soluble Materials

Andrea Camposeo, Francesca D'Elia, Alberto Portone, Francesca Matino, Matteo Archimi, Silvia Conti, Gianluca Fiori, Dario Pisignano, and Luana Persano**

Supporting Information

Naturally-Degradable Photonic Devices with Transient Function by Heterostructured Waxy-Sublimating and Water-Soluble Materials

Andrea Camposeo, Francesca D'Elia, Alberto Portone, Francesca Matino, Matteo Archimi, Silvia Conti, Gianluca Fiori, Dario Pisignano, Luana Persano**

Dr. A. Camposeo, Dr. A. Portone, Dr. F. Matino, Dr. M. Archimi, Prof. D. Pisignano, Dr. L. Persano
NEST, Istituto Nanoscienze-CNR, Piazza S. Silvestro 12, I-56127 Pisa, Italy
E-mail: luana.persano@nano.cnr.it
Dr. A. Camposeo, F. D'Elia, Dr. A. Portone, Dr. F. Matino, Dr. L. Persano
NEST, Scuola Normale Superiore, Piazza S. Silvestro 12, I-56127 Pisa, Italy
Dr. S. Conti, Prof. G. Fiori
Dipartimento di Ingegneria dell'Informazione, Università di Pisa, Via Caruso 16, I- 56122 Pisa, Italy
Dr. M. Archimi, Prof. Dario Pisignano
Dipartimento di Fisica, Università di Pisa, Largo B. Pontecorvo 3, I-56127 Pisa, Italy
E-mail: dario.pisignano@unipi.it

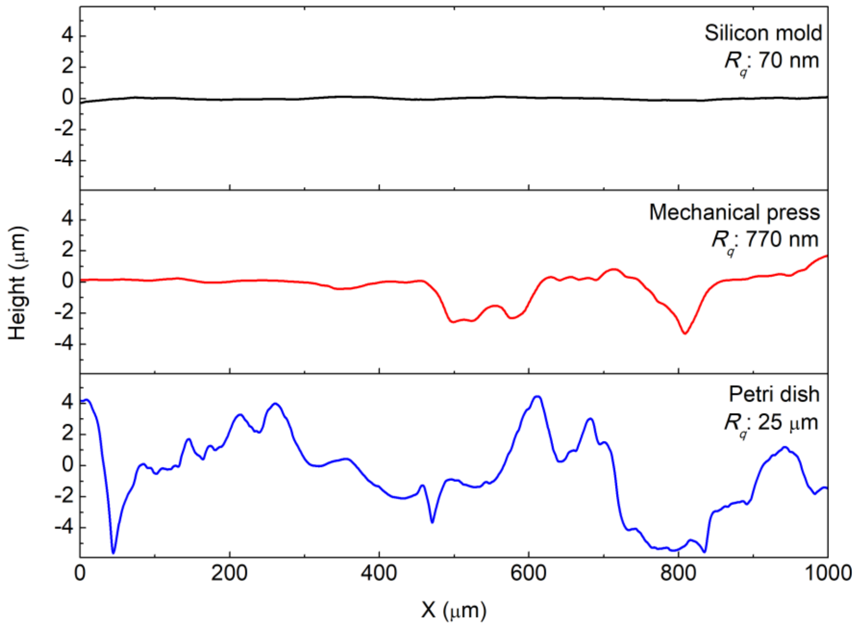

Figure S1. Height profiles of CDD surfaces upon molding by different techniques. From top to bottom: Heating-cooling cycles in pre-formed silicon molds, mechanical pressing of solid grains, heating-cooling in Petri dishes. R_q: calculated root mean square roughness.

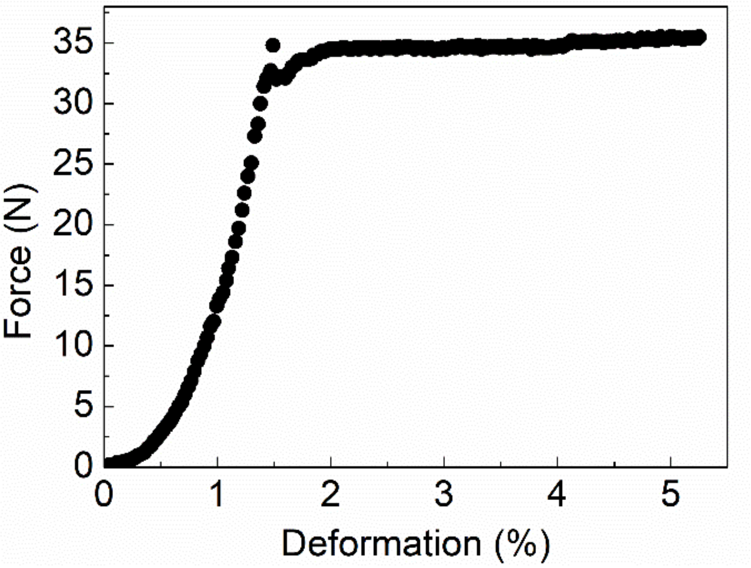

Figure S2. Force-deformation plot of a CDD substrate measured at 26°C under compression. Compression rate: 0.5 mm/min.

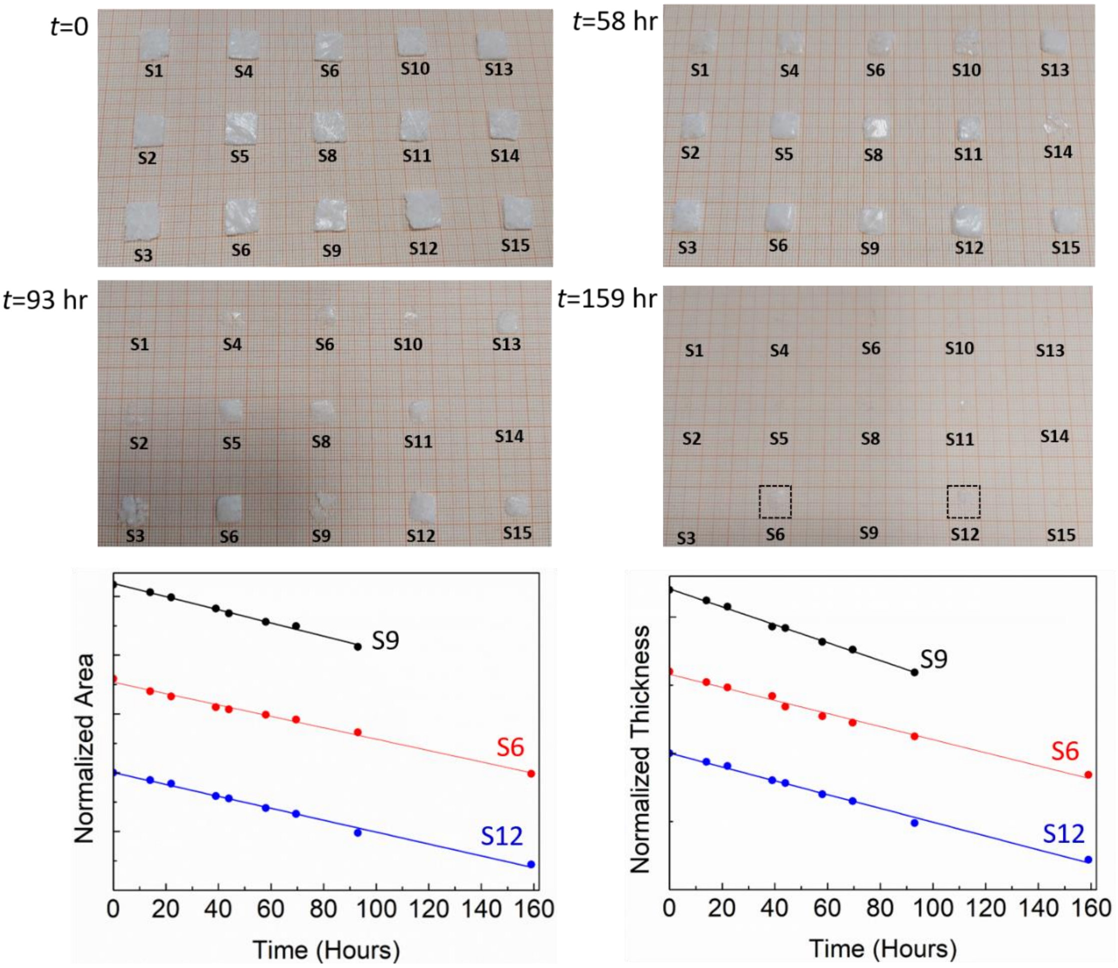

Figure S3. Top photographs: CDD substrates (S1-S15) captured at different instant times (*t*=0, 58, 93, 159 hours) during sublimation at room temperature under a fume hood with a face velocity of 1.9 m s^{-1}. Bottom plots show the normalized area (left) and thickness (right) *vs*. sublimation time, for the samples labelled by S6, S9 and S12 (data vertically-shifted for better clarity).

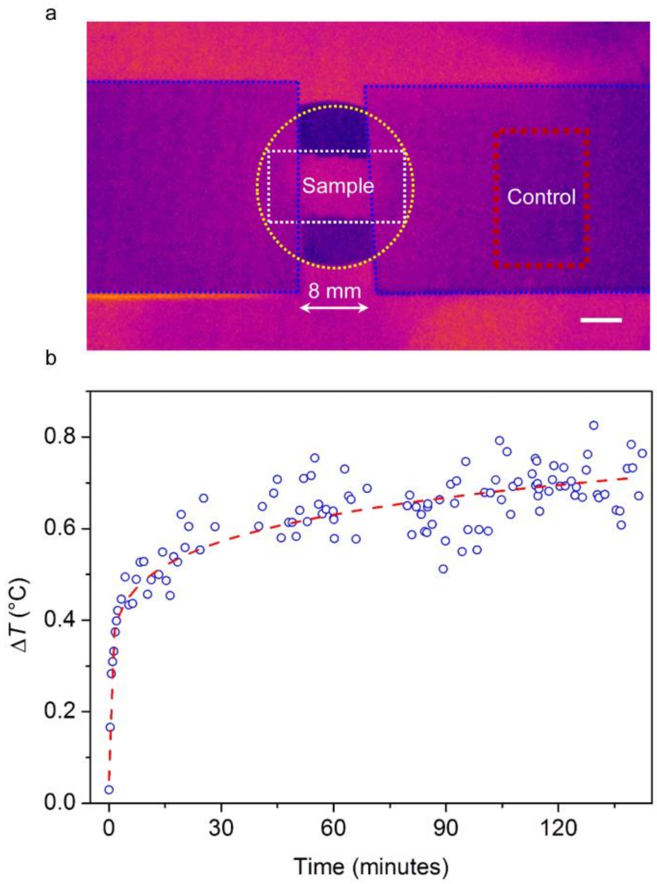

Figure S4. (a) Infrared thermal image of a CDD sample (white box), illuminated under an optical microscope (illuminated region: yellow dashed circle). The sample is suspended between two glass slides, highlighted by blue dashed lines. A second CDD sample (red box), positioned in a region that is not illuminated, serves as reference for determining the illumination-induced temperature variation (ΔT). This thermal photograph is collected after 15 minutes of continuous irradiation. Scale bar: 5 mm. (b) Temporal evolution of ΔT during illumination (circles), evidencing an overall temperature increase below 1°C. The dotted line is a guide for the eye.

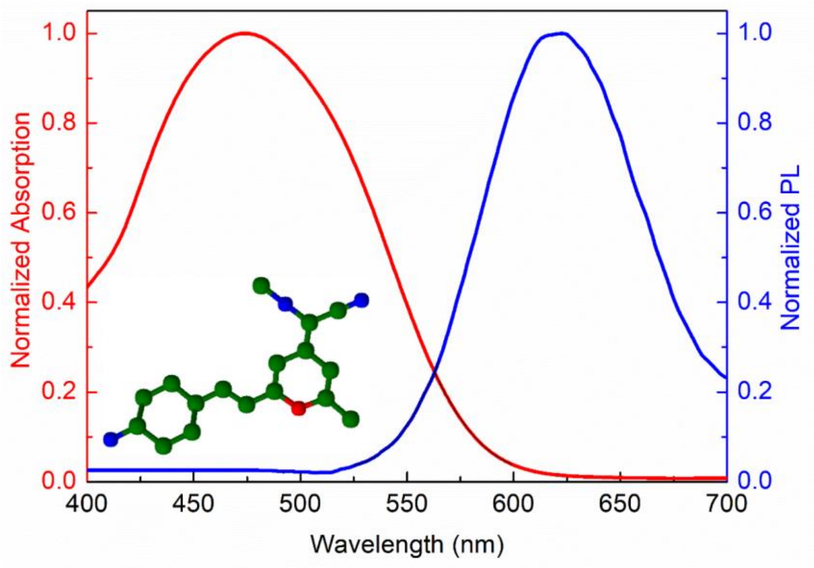

Figure S5. Absorption (red line, left vertical scale) and PL (blue line, right vertical scale) spectra of a PVA/DCM:PVP bilayer deposited on a quartz substrate. Dye-doping in PVP is carried out at 1% wt:wt DCM:PVP concentration. Inset: chemical structure of DCM (Colours indicate different atoms. Red: oxygen, green: carbon, blue: nitrogen).

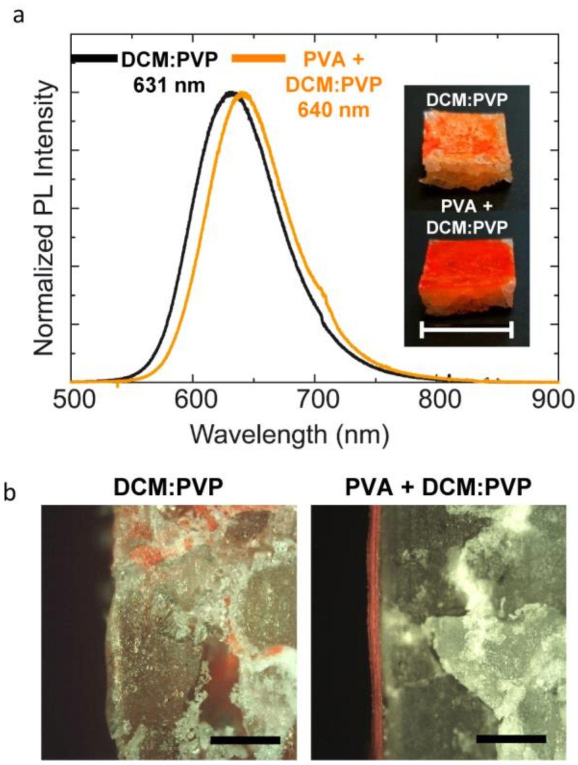

Figure S6. Comparison between films of PVP doped with 1% wt:wt of DCM (DCM:PVP) deposited either directly on top of CDD or on an intermediate PVA layer. (a) PL and photographs of the samples highlighting a blue-shift of the emission and a permeation of DCM:PVP into the CDD in absence of the PVA. Scale bar: 1 cm (b) Optical micrographs of the sample cross sections, supporting DCM:PVP permeation in the substrate without PVA, and showing the planarizing effect of the PVA coating. Scale bar: 400 μm.

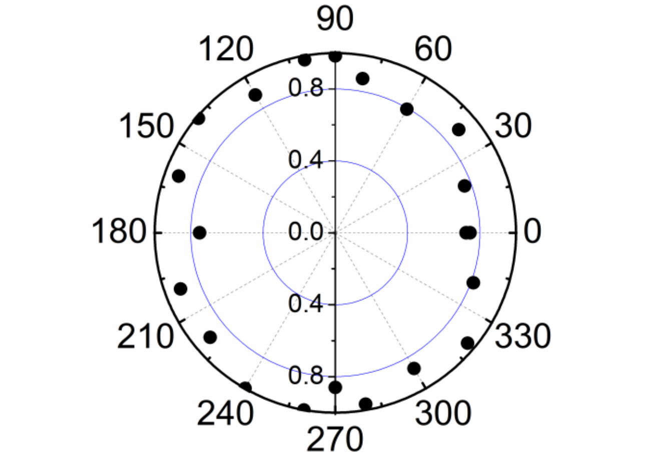

Figure S7. Polar plot of the normalized ASE intensity (circles) as a function of the angle of the analyzer polarization filter axis, θ, measured with respect to the device thickness axis (θ = 0° for polarizer axis parallel to the device thickness axis, θ = 90° for polarizer axis perpendicular to the device plane).

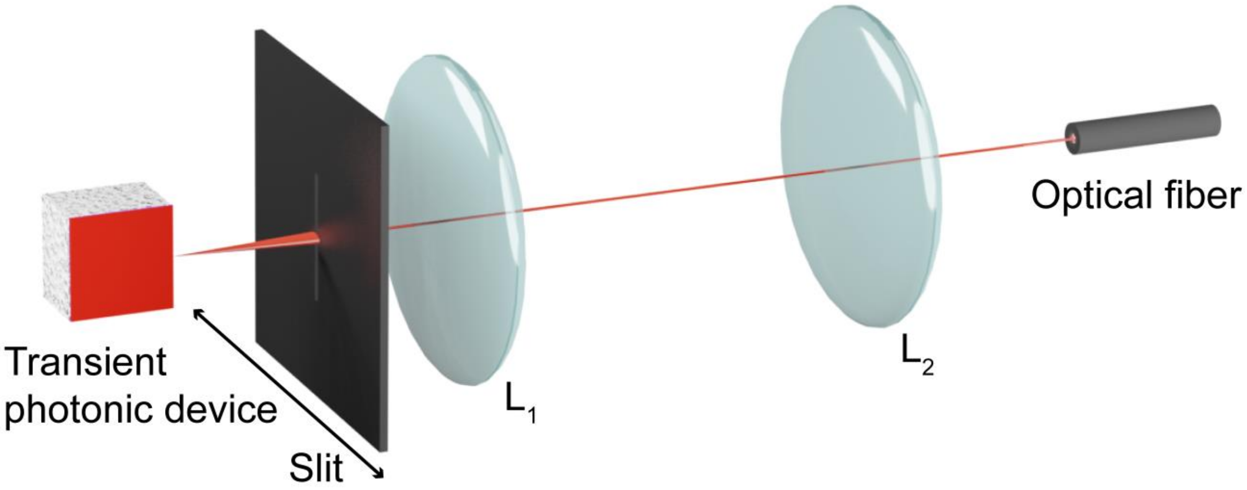

Figure S8. Experimental geometry used for measuring the ASE beam divergence.

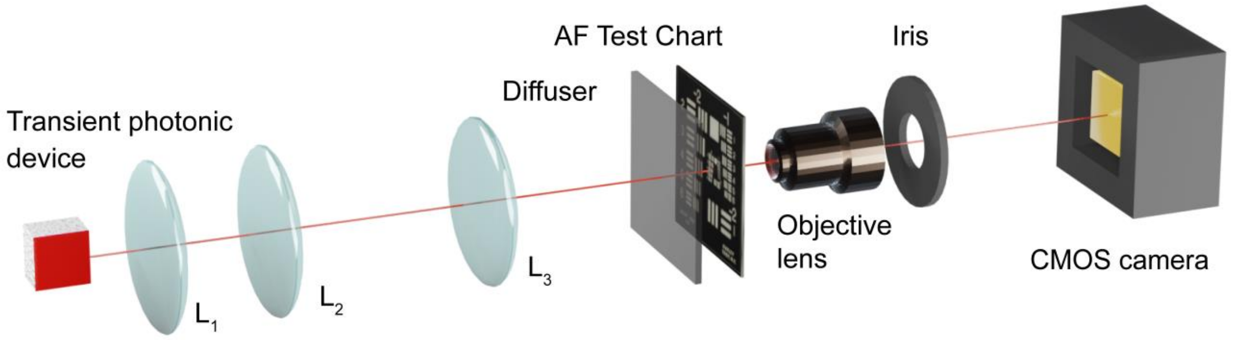

Figure S9. Scheme of the experimental set-up used for full-field imaging experiments.

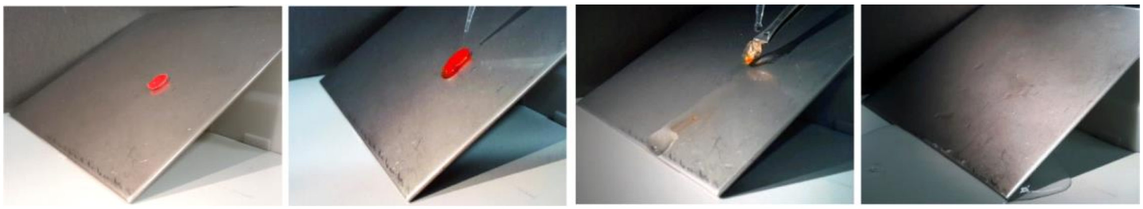

Figure S10. Photographs of dissolution of the residual polymer layers in water, at representative instants.
